# Recent progress in the development of DNA-based biosensors integrated with hybridization chain reaction or catalytic hairpin assembly

**DOI:** 10.3389/fchem.2023.1134863

**Published:** 2023-02-16

**Authors:** Liuting Mo, Wanqi He, Ziyi Li, Danlian Liang, Runhong Qin, Mingxiu Mo, Chan Yang, Weiying Lin

**Affiliations:** Guangxi Key Laboratory of Guangxi Key Laboratory of Electrochemical Energy Materials, School of Chemistry and Chemical Engineering, Institute of Optical Materials and Chemical Biology, Guangxi University, Nanning, China

**Keywords:** hybridization chain reaction, catalytic hairpin assembly, biosensor, disease diagnosis, cell imaging

## Abstract

As isothermal, enzyme-free signal amplification strategies, hybridization chain reaction (HCR) and catalytic hairpin assembly (CHA) possess the advantages such as high amplification efficiency, excellent biocompatibility, mild reactions, and easy operation. Therefore, they have been widely applied in DNA-based biosensors for detecting small molecules, nucleic acids, and proteins. In this review, we summarize the recent progress of DNA-based sensors employing typical and advanced HCR and CHA strategies, including branched HCR or CHA, localized HCR or CHA, and cascaded reactions. In addition, the bottlenecks of implementing HCR and CHA in biosensing applications are discussed, such as high background signals, lower amplification efficiency than enzyme-assisted techniques, slow kinetics, poor stability, and internalization of DNA probes in cellular applications.

## 1 Introduction

**FIGURE 1:**
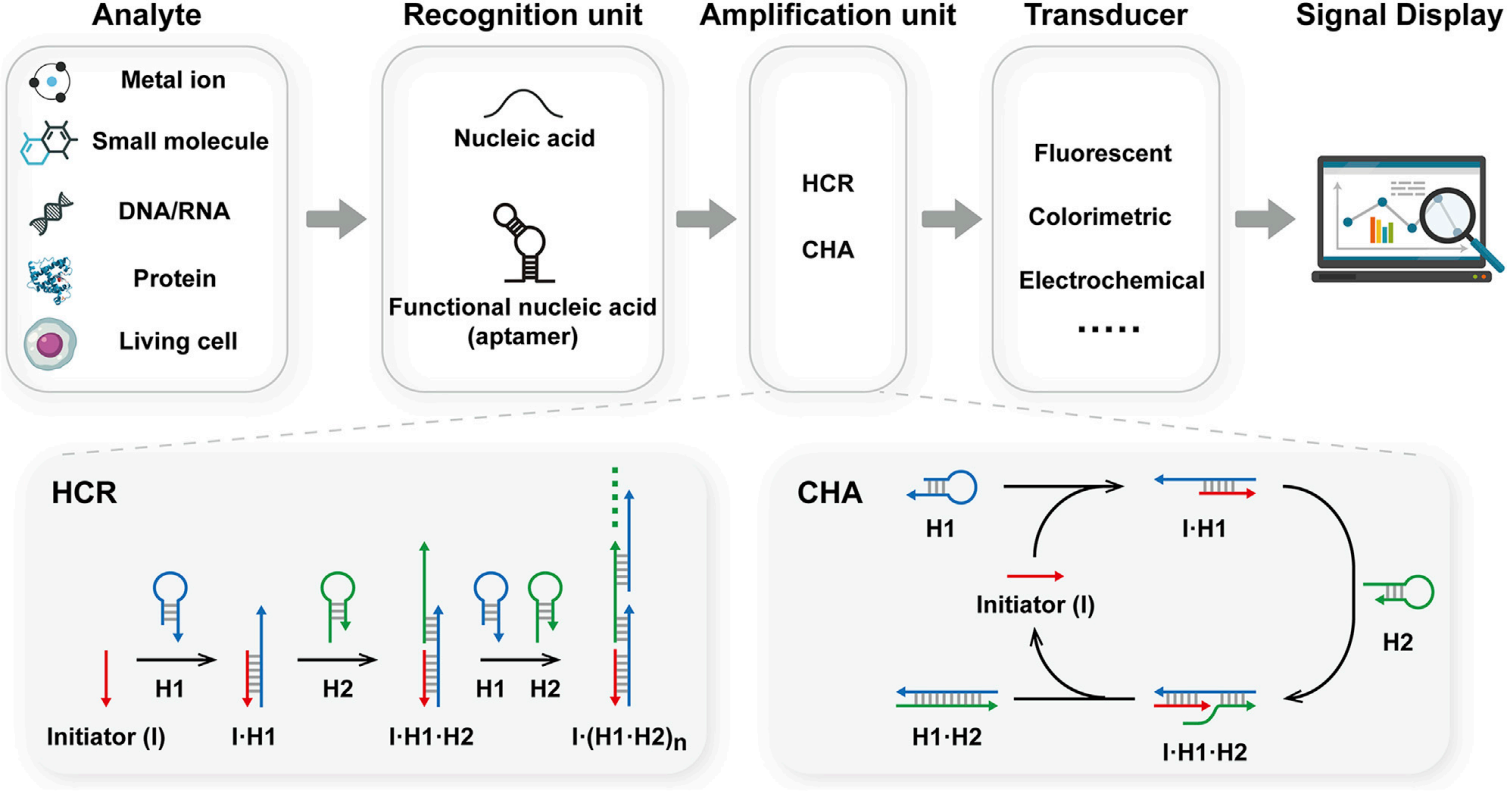
Schematic illustration of the construction of DNA-based biosensors integrated with HCR or CHA.

Due to their high programmability, stability, and biocompatibility, nucleic acids are popular in the development of dynamic circuits and nanostructures (Zhang et al., 2014; Sadowski et al., 2014; Wang et al., 2017). To date, various DNA-based biosensors have been reported for bioanalytical applications (Yuan et al., 2018; Mo et al., 2023; Mo et al., 2021). A DNA-based biosensor consists of three main components: a recognition unit, an amplification unit, and a transducer (Figure 1). The recognition unit is used to interact with analytes. In which target DNA/RNA could be recognized *via* Watson-Crick base pairing. Moreover, the development of aptamers has expanded the scope of analytes to include metal ions, small molecules, proteins, and living cells (Hansen-Bruhn et al., 2018; Qin et al., 2019; Song et al., 2022). Upon binding to the target, the recognition unit undergoes a conformational change and exposes the trigger domain for the amplification unit, which then initiates the amplification process. Various amplification techniques have been developed to enhance sensitivity, including HCR and CHA (Cheglakov et al., 2015; Chai and Miao, 2019; Wang et al., 2021). These amplification strategies could be divided into two categories: enzyme-assisted amplification and enzyme-free amplification. The first category relies on the catalytic capability of enzymes to realize nucleic acid amplification, such as polymerase chain reaction (PCR) (Saiki et al., 1988) and rolling circle amplification (RCA) (Zhao et al., 2008). While the sensitivity could be greatly improved, the use of enzymes has limited their application in complex environments. On the other hand, enzyme-free amplification, such as hybridization chain reaction (HCR) (Dirks and Pierce, 2004) and catalytic hairpin assembly (CHA) (Yin et al., 2008), could amplify nucleic acids through toehold-mediated strand displacement. Notably, these methods could operate under mild and isothermal conditions, without the involvement of enzymes or thermal cyclers. These features make them suitable for applications in biological environments, especially in living cells. The isothermal, enzyme-free amplification techniques, HCR and CHA, provide high amplification efficiency, high biocompatibility, ease of operation, and low cost. In addition, several advanced techniques have recently been reported to improve performance, such as branched HCR or CHA, and localized HCR or CHA. The last component of the biosensor was the transducer, which transformed the nucleic acid information into a measurable signal, such as fluorescent, colorimetric, and electrochemical signals (Huang et al., 2020; Sun et al., 2021). In this review, we will summarize the research progress on biosensors based on HCR and CHA, as well as related advanced amplification methods, with particular attention to sensing platforms based on pure DNA materials, such as nucleic acid circuits or DNA nanostructures. The challenges encountered in the development of sensors will also be discussed. We hope this paper will provide updated information for sensing strategies based on HCR and CHA, and facilitate their applications in bioanalysis, clinical diagnosis, and environmental monitoring.

## 2 DNA-based biosensors integrated with HCR or CHA

### 2.1 Biosensors based on traditional or advanced HCR

#### 2.1.1 Traditional HCR

**FIGURE 2:**
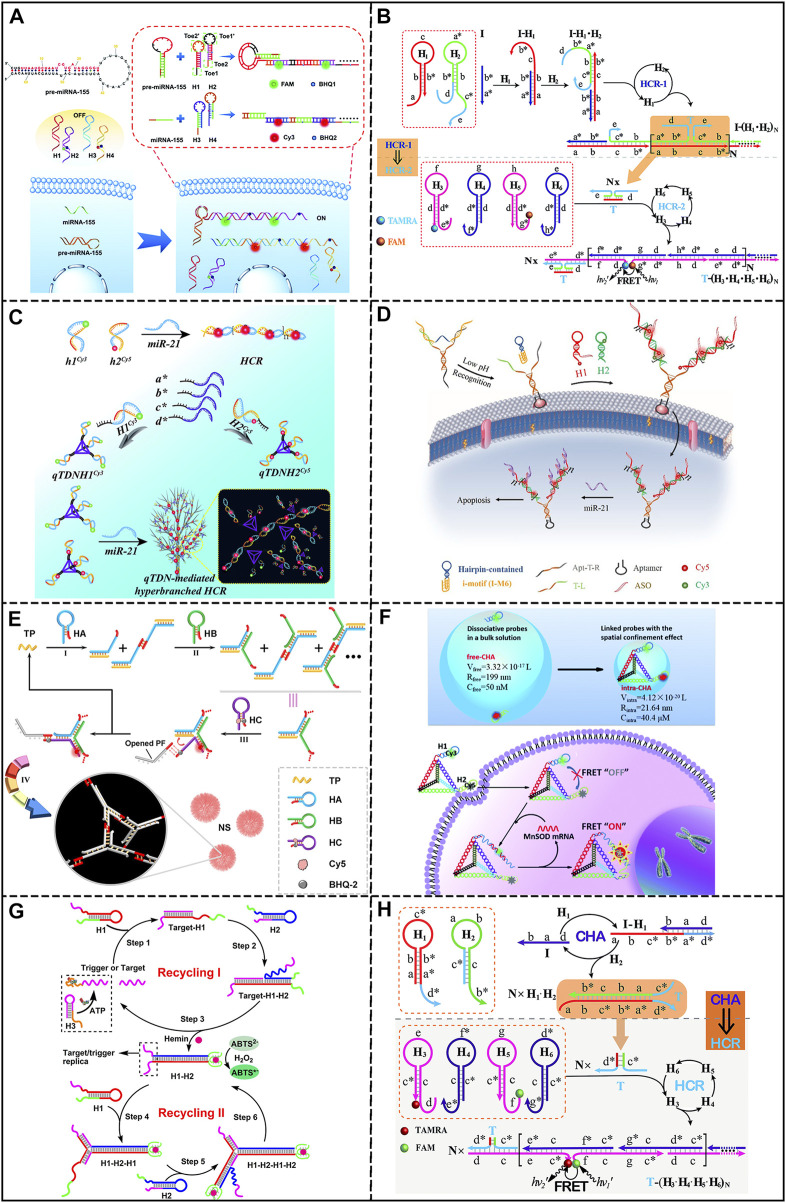
DNA-based biosensors integrated with HCR or CHA. **(A)** Biosensor based on traditional HCR. Reproduced from (Yang et al., 2019) with permission from the Royal Society of Chemistry. **(B)** Biosensor based on branched HCR. Reproduced from (Wei et al., 2018a) with permission from the Royal Society of Chemistry. **(C)** Biosensor based on localized HCR. Reproduced from (Wang et al., 2019a) with permission from the Royal Society of Chemistry. **(D)** Biosensor based on other HCR. Reproduced from (Ma et al., 2019) with permission from the American Chemical Society. **(E)** Biosensor based on branched CHA. Reproduced from (Liu et al., 2020) with permission from the American Chemical Society. **(F)** Biosensor based on localized CHA. Reproduced from (Qing et al., 2020) with permission from the Royal Society of Chemistry. **(G)** Biosensor based on self-replicating CHA. Reproduced from (Dai et al., 2017) with permission from the American Chemical Society. **(H)** Biosensor based on cascaded CHA-HCR strategy. Reproduced from (Wang et al., 2018) with permission from the Royal Society of Chemistry.

Classical HCR was firstly developed by Dirks and Pierce (Dirks and Pierce, 2004), which involves an initiator and two metastable hairpins. The initiator could trigger the alternate opening of hairpins, forming nicked double-stranded DNA structures. As a simple and powerful amplification method, HCR has been incorporated into a number of sensing platforms. A high-fidelity amplified FISH system was reported by Marras et al. (2019), in which the target mRNA interacted with the binary probe to release the HCR initiator. This system could distinguish single nucleotide variations within a single mRNA. In another study, Yang et al. (2019) realized the simultaneous detection of miRNA and pre-miRNA in living cells through two pairs of HCR hairpin probes, showing detection limits of 680 pM and 820 pM, respectively (Figure 2A). Later, Tang et al. (2020) integrated HCR with the dual-split aptamer probe (DSAP) for activatable cancer cell detection. In this design, DSAP could specifically recognize cancer cells and release the trigger for HCR, leading to *in-situ* HCR on the cell membrane and amplified fluorescence. This strategy could reach a detection limit of 20 cells/200 uL. Overall, these studies proved the potential of HCR in biosensing and bioimaging.

#### 2.1.2 Branched HCR

Branched hybridization chain reaction (bHCR) involves hierarchical reactions of HCR, producing branched DNA polymers and higher levels of amplification efficiency. For example, Wang’ group (Wei et al., 2018a) developed a concatenated HCR system for miRNA detection. As shown in Figure 2B, target RNA could trigger the first layer of HCR, exposing the initiator for the second layer of HCR, resulting in a shortened distance between FRET donor and acceptor. The detection limit was calculated to be 3 pM. Soon after, the same group integrated the concept of cascade HCR with logic gate operation for intracellular miRNA imaging. In this study, the presence of miRNA was transduced to the initiator of cascading HCR through biocomputing operations. By rationally designing the sensing module, binary or advanced logic gates were achieved. (Gong et al., 2019). To obtain better sensitivity, electrochemical detection has also been implemented with branched HCR for miR-141 detection, achieving a LOD of 0.18 fM (Li et al., 2019). Additionally, by integrating aptamers into the branched HCR system, biomolecules, such as ATP, can be detected. In a study by Zhou et al. (2019), the binding of ATP aptamer to the target released the initiator for branched HCR, leading to FRET signals with a detection limit of 170 nM.

#### 2.1.3 Localized HCR

The kinetics of typical HCR is limited since the working principle is based on random collision of hairpins. To accelerate the reaction, localized HCR (LHCR) was developed based on the spatial-confinement effect. The reaction time could be reduced by anchoring the reactants to DNA nanostructures in order to reach higher local concentration of hairpins. For instance, Bui et al. (2017) immobilized the HCR hairpins along a 1D linear DNA track, which enabled the hairpins to hybridize along the track. The kinetics of this strategy was six times faster than traditional HCR due to the locality effect. To further apply the concept of LHCR to sensing applications; Wu et al. (2020a) developed a tripartite Y-shaped DNA probe for mRNA imaging in living mice. Y-shaped scaffolds were functionalized with HCR hairpins and a folate (FA) ligand that could selectively enter tumor cells, leading to HCR assemblies on the scaffold in response to miR-21. Moreover, taking advantage of the stability of Y-shaped structure, the probe was successfully applied for high-contrast miRNA imaging in living mice. In addition, 3D DNA nanostructures have also been employed for LHCR. As illustrated in Figure 2C, Wang et al. (2019a) reported hyperbranched HCR mediated by tetrahedral DNA nanostructures (qTDN). By modifying HCR hairpins on the vertices of qTDN, the reaction orientation was extended, as well as the collision probability and reaction kinetics. The reaction rate is about 70 times faster than that of traditional HCR, and the detection limit was 2.14 pM. These reports confirmed the acceleration effect of LHCR.

#### 2.1.4 Other HCR

In addition to the strategies mentioned above, other HCR technologies have been developed. A recent study by Cheng et al. (2021) used proximity-dependent surface hybridization chain reactions to design an electrochemical biosensor for miRNA detection. MiRNA triggered HCR and the formation of duplexes with single-stranded dangling tails, which were labeled with ferrocene (Fc) and could be used to identify the sequence fixed on the electrode. After identification, the Fc tags were brought proximal to the electrode, which induced amplified electrochemical signals with a LOD of 3 fM. Furthermore, Jiang et al. (2021) presented a dumbbell hybridization chain reaction (DHCR), which improved the amplification performance by a novel morphology of the DNA nanostructure. This system consisted of four probes (A, B, C, D), in which probes A and B contained PSA split aptamers that could recognize PSA. However, in the absence of PSA, the unhybridized parts of probes A and B could alternatively open dumbbell probes C and D, forming a stacked DNA nanostructure and enhancing electrochemical signals. The LOD for PSA was 0.5 pg/mL. Moreover, HCR can be regarded as an ideal choice for target cell imaging and gene delivery. Ma et al. (2019) reported a bipedal hybridization chain (BiHCR) for activable cancer cell imaging (Figure 2D). A Y-shaped nanoprobe was constructed containing an i-motif sequence, an aptamer sequence and two trigger strands for HCR. After specific binding between the aptamer and cancer cells, the acidic extracellular environment triggered a conformational change in the i-motif structure, exposing the HCR initiators. Which further induced *in-situ* bipedal HCR and produced FRET signals. In addition, Peng et al. (2022) developed a double loop-stem mediated HCR (D-HCR) for label-free biosensing. The presence of miR-21 not only triggered the formation of DNA nanowires, but also released previously locked G4 aptamers. The LOD of 4.60 pM was achieved by combining D-HCR with thioflavin T (ThT) for fluorescent detection, and 13.60 pM was achieved by incorporating Hemin for colorimetric detection.

### 2.2 Biosensors based on traditional or advanced CHA

#### 2.2.1 Traditional CHA

The CHA was firstly introduced by Yin’s group (Yin et al., 2008), in which an initiator could catalyze the duplex formation between two metastable hairpins, resulting in the amplification of double-stranded DNA products. Owing to the feature of simple operation, mild conditions, and flexibility, CHA has been widely applied in the development of sensors. For example, a universal CHA system (uniCHA) was proposed by Zhang et al. (2022) to detect various piRNAs and miRNAs. An adaptable starting hairpin containing the CHA initiator was employed in this system. The targets would interact with the starting hairpin to expose the initiator, which could trigger CHA and generate FRET signals. It is noteworthy that the sequence of the starting sequence can be adjusted depending on the target. Therefore, this system has been applied to the detection of various piRNAs and miRNAs. In addition, CHA can be integrated with lateral flow immunoassay (LFIA) to detect SARS-CoV-2 RNA (Zou et al., 2021). In this study, target RNA catalyzed the formation of a H1-H2 duplex, which was then detected by LFIA *via* digoxin/biotin recognition. The detection limit was calculated to be 2000 copies/mL. These studies demonstrate the potential of CHA for clinical diagnosis.

#### 2.2.2 Branched CHA

Branched CHA (bCHA) usually employ three or more hairpin probes to form three-way or four-way junctions upon the initiator. Due to the higher energy barriers, bCHA had a much lower background signal than traditional CHA, providing higher detection sensitivity. Yue et al. (2019) employed bCHA for miRNA imaging in living cells by anchoring three CHA hairpins on a Y-shaped DNA scaffold. After entering the cell, the probe could undergo bCHA through the catalysis of miR-155, leading to the formation of DNA dendrimers and *in-situ* imaging of miRNA. In another study, Liu et al. (2020) proposed a miRNA imaging method based on *in-situ* growth of DNA nanostructures by introducing palindromic sequences to bCHA hairpins. According to Figure 2E, upon miR-21, bCHA could be triggered to form Y-shaped structures, which could further assemble into 3D nanospheres owing to the palindromic fragments at the end of the hairpins. The process of bCHA also caused separation of fluorophore and quencher, leading to the recovery of fluorescence and reaching a LOD of 1.4 pM. Other than miRNA detection, bCHA has also found its application in antibiotic detection, Zhang et al. (2019) developed an aptasensor for the simultaneous detection of chloramphenicol (CAP) and kanamycin (Kana). In the presence of CAP or Kana, CHA hairpins can be catalyzed to form three-arm or four-arm branched junctions, which could be distinguished by microchip electrophoresis (MCE) based on migration time. And the detection limits for CAP and Kana were 0.49 pg/mL and 0.52 pg/mL, respectively.

#### 2.2.3 Localized CHA

Localized CHA (LCHA) takes advantage of the spatial-confinement effect by limiting CHA hairpins in a compact area, leading to higher amplification efficiency and reaction rate. Similar to LHCR, the hairpins could be fixed on 1D, 2D, or 3D DNA nanostructures to realize LCHA, such as DNA origami (Dai et al., 2021), Y-shaped scaffolds (Wu et al., 2020b), DNA nanocubes (Liu et al., 2019; Feng et al., 2022), DNA triangular prisms (DTP) (Yang et al., 2020), and DNA nanolanterns (Wang et al., 2022a). Recently, a nanowire-based LCHA for miRNA imaging was reported by Wei et al. (2018b). Target RNA catalyzed the hybridization between adjacent hairpins on the nanowire, leading to the recovery of quenched fluorescence. The LOD of this method was 2.0 pM, which was about 11.8 times lower than typical CHA. In addition, Bai et al. (2022) developed a dispersion-to-localization of catalytic hairpin assembly (DL-CHA) strategy. In the presence of miR-21, CHA was induced between hairpins anchored to linker strands, which promoted the formation of DNA ladders. A high signal-to-background (S/B) ratio was reached at 37.8, with a wide linear range of 100 pM to 100 nM. Moreover, Qing et al. (2020) developed an intramolecular CHA strategy for mRNA imaging, where the distance between reactants were shortened by anchoring the CHA hairpins on a DNA tetrahedron (Figure 2F). The local concentration of hairpins was about 808 times higher than free CHA, which increased the initial reaction rate by 15.6 times. Later, an intermolecular CHA strategy for FRET imaging of miRNA-155 in cells was proposed by Wang et al. (2022b). By anchoring CHA hairpins on separate DNA tetrahedrons, the targets could trigger CHA between DNA nanostructures, resulting in polymerized products and amplified FRET signals.

### 2.3 Biosensors based on cascaded reaction

For the development of biosensors with higher sensitivity, multiple amplification strategies could be cascaded, whereby the product of the upstream reaction acts as a trigger for the downstream reaction. In this section, we will discuss recent advances in biosensors based on cascade reactions involving HCR and CHA.

#### 2.3.1 Cascaded reaction of CHA and CHA

Cascaded CHA could achieve exponential signal growth by interconnecting multiple CHA circuits. For example, Guk et al. (2019) reported a self-circulation CHA system for miRNA detection. According to their design, miR-34a initiated the first CHA cycle and released the catalyst for the second CHA cycle, which in turn produced the initiator for the first cycle. Owing to its versatility, this strategy has been adapted for interferon-γ (IFN-γ) detection, reaching a LOD of 0.6 pM (Wang et al., 2019b). Besides, another promising approach is the self-replicating catalytic hairpin assembly system (SRCHA), in which only two hairpins were required for the cascade reaction. As illustrated in Figure 2G, the target could trigger the first CHA cycle and generate duplexes with exposed trigger sequences. Therefore, the CHA product may serve as a new CHA initiator. This system has been successfully applied to the detection of metal ions, biomolecules, and nucleic acids (Dai et al., 2017; Pan et al., 2019).

#### 2.3.2 Cascaded reaction of CHA and HCR

The cascaded integration of CHA and HCR is expected to provide higher amplification efficiency than single amplification. As shown in Figure 2H, Wang et al. (2018) established a general CHA-HCR sensing platform, where the outputs of CHA induced by the target miRNA could serve as the initiators for HCR circuits, showing a LOD of 2 pM. Later, Li et al. (2022) integrated the concept of palindrome-based HCR (PHCR) with CHA for miR-21 detection. The presence of miR-21 catalyzed the formation of CHA products, which further initiated HCR between hairpins with palindromic domains, leading to the formation of crosslinking networks through the assembly of HCR products. With amplified FRET signals, target RNA could be detected down to 10 pM. The strategy of HCR-CHA has also been employed in sensors. Wei et al. (2016) integrated split CHA initiators into HCR hairpins. The presence of miR-144 could trigger HCR and co-localize the split sequences, which further initiated the CHA process and induced amplified fluorescence, reaching the LOD of 0.3 fM.

## 3 Conclusions and perspectives

The last decade has seen a rapid development in HCR and CHA for the biosensing applications. Based on their features of being isothermal and enzyme-free, these techniques have been employed for the detection of metal ions, small molecules, proteins, cells, tissues and living animals.

Despite significant progress, the applications of isothermal and enzyme-free amplification still face several challenges. 1) To improve detection accuracy, the problem of background leakage must be resolved. During the spontaneous breathing process of DNA duplexes, non-specific reactions between hairpins of HCR or CHA may occur in the absence of the initiator, leading to a high background signal. To minimize background signals, more effort should be directed toward rationally designing the sequence in order to avoid unexpected reactions. 2) In terms of sensitivity, traditional HCR or CHA showed limited amplification efficacy compared to enzyme-assisted amplification. To increase sensitivity, the development of cascaded amplification strategies would be beneficial (Wang et al., 2018; Wang et al., 2019b). 3) The kinetics are relatively slow since typical HCR and CHA rely on random collisions of hairpins. The strategy of localized HCR or CHA could provide a promising method to achieve a faster reaction time by exploiting the spatial-confinement effect (Wang et al., 2019a; Qing et al., 2020). 4) The majority of reported strategies focus on singlexed detection. However, it is necessary for clinical diagnosis of diseases to determine the level of multiple markers. Thus, constructing multiplexed sensing platform is more beneficial for biomedical applications. However, the sequence of hairpins needs to be designed for different targets, thereby limiting their applications in multi-target analysis. Fortunately, several universal sensing systems have been proposed, where the initiator was embedded in a starting hairpin. By adjusting the sequence of the starting hairpins according to the target, the sequence of the remaining hairpins could remain unchanged (Huang et al., 2016; Zhang et al., 2022). 5) The cellular delivery of nucleic acid probe remains a challenging task. Luckily, the development of DNA nanotechnology has offered a bunch of nanocarriers with controllable geometry, easy synthesis, and high biocompatibility. Integrating HCR or CHA hairpins on DNA nanostructures is a promising approach for achieving intracellular internalization (Liang et al., 2014; Raniolo et al., 2019; Peng et al., 2021). 6) The stability of nucleic acid probes is a major concern, especially in real samples, such as food or blood. There has been evidence that DNA nanocarriers can be effective at increasing stability (Li et al., 2021). Alternatively, there is the option of using artificial nucleic acids, such as L-DNA, peptide nucleic acids, and locked nucleic acids (Olson et al., 2017).

In spite of the challenges, continuous attention has been paid to the development of isothermal, enzyme-free amplification techniques. It is believed that more advanced amplification strategies based on HCR and CHA will be emerging, and more targets will be detected with the aid of aptamers or antibodies, and more signal transduction methods (e.g., mass spectrometry, ultrasound imaging, magnetic resonance imaging) may be integrated to develop multifunctional sensors. Eventually, these efforts will result in more useful tools for bioanalysis, clinical diagnosis, and environmental monitoring.
